# The triterpenoids of *Hibiscus syriacus* induce apoptosis and inhibit cell migration in breast cancer cells

**DOI:** 10.1186/s12906-015-0592-9

**Published:** 2015-03-14

**Authors:** Ren-Jun Hsu, Yao-Chin Hsu, Shu-Pin Chen, Chia-Lynn Fu, Jyh-Cherng Yu, Fung-Wei Chang, Ying-Hsin Chen, Jui-Ming Liu, Jar-Yi Ho, Cheng-Ping Yu

**Affiliations:** Department of Pathology, and Graduate Institute of Pathology and Parasitology, Tri-Service General Hospital, National Defense Medical Center, Taipei, Taiwan; Biobank Management Center of Tri-Service General Hospital, National Defense Medical Center, Taipei, Taiwan; Departments of Chinese Medicine, Chi-Mai Medical Center, Tainan, Taiwan; Division of Medical Genetics, Department of Pediatrics, Chang Gung Children’s Hospital, Taoyuan, Taiwan; Department of General Surgery, Tri-Service General Hospital, National Defense Medical Center, Taipei, Taiwan; Department of Obstetrics & Gynecology, Tri-Service General Hospital, National Defense Medical Center, Taipei, Taiwan; Department of Emergency Medicine, Tri-Service General Hospital, National Defense Medical Center, Taipei, Taiwan; Division of Urology, Department of Surgery, Taoyuan General Hospital, Ministry of Health and Welfare, Taoyuan, Taiwan; Graduate Institute of Life Sciences, National Defense Medical Center, Taipei, Taiwan

**Keywords:** *Hibiscus syriacus*, Betulin, Breast cancer

## Abstract

**Background:**

Breast cancer-related mortality increases annually. The efficacy of current breast cancer treatments is limited, and they have numerous side effects and permit high recurrence. Patients with estrogen receptor (ER)-negative or triple-negative breast cancer are particularly difficult to treat. Treatment for this type of cancer is lacking, and its prognosis is poor, necessitating the search for alternative treatments.

**Methods:**

This study screened Chinese herb *Hibiscus syriacus* extracts and identified a novel anti-cancer drug for patients with ER-negative breast cancer. The inhibitory effects on cell viability and migration were evaluated for each compound, and the molecular regulatory effects were evaluated on both mRNA and protein levels.

**Result:**

We found several triterpenoids including betulin (K02) and its derivatives (K03, K04, and K06) from *H. syriacus* inhibited human triple-negative breast cancer cell viability and migration but revealed smaller cytotoxic effects on normal mammalian epithelial cells. Betulin and its derivatives induced apoptosis by activating apoptosis-related genes. In addition, they activated p21 expression, which induced cell cycle arrest in breast cancer cells. Betulin (K02) and betulinic acid (K06) had stronger inhibitory effects on cell viability and migration than K03 and K04.

**Conclusions:**

*H. syriacus* extracts might inhibit breast cancer cell viability and induce apoptosis by activating p53 family regulated pathways and inhibiting AKT activation. *H. syriacus* extracts may provide important insight into the development of novel alternative therapies for breast cancer.

**Electronic supplementary material:**

The online version of this article (doi:10.1186/s12906-015-0592-9) contains supplementary material, which is available to authorized users.

## Background

Breast cancer is one of the most common cancers in females worldwide. Approximately 500,000 people die annually from breast cancer, and about one out of eight females are diagnosed with breast cancer [1]. Currently, the main treatment for breast cancer is surgery, along with radiotherapy, chemotherapy, hormone, and immunological treatment. The efficacy of current treatments for breast cancer is limited. In addition to side effects, the risk of recurrence is high. Clinically, 70% of breast cancer patients are positive for the estrogen receptor (ER) in the tumor. After treatment with tamoxifen, the death rate and recurrence rate is reduced by 50% and 25%–30%, respectively. However, one-third of patients showed recurrence within fifteen years after tamoxifen treatment, and the cancer cells’ failure to express estrogen receptor (ER) is one of the most important reasons [2]. Furthermore, 10%–15% of breast cancers are of the triple-negative subtype. Treatment is lacking for patients with this type of breast cancer, and their prognosis is poor [3]. Thus, treatment for breast cancer has been a difficult issue.

Many patients seek natural, traditional treatments to alleviate or improve breast cancer and the side effects caused by treatment. Herbal therapy has been used for thousands of years, and there are several organisms that provide a source of anti-cancer drugs in nature. Several Chinese herbal medicines are effective against diseases, but most of the effects and mechanisms of action are not scientifically supported. The effect of Chinese herbal medicines on cancer has become a hot research topic. For instance, EGCG extracted from green tea inhibits the growth of pancreatic cancer cells [4]. EGCG also induces MCF-7 cells towards p53-related apoptosis in breast cancer cells [5]. Intake of EGCG extracted from green tea reduces the occurrence of head and neck cancer [6]. Besides, huanglian increases the inhibition of cell cycle-related protein expression, leading to suppressed growth of cancer cells [7]. The popular sporophore extract from *Antrodia cinnamomea* reduces the invasive ability of hepatoma cells by inhibiting the NF-κB signal transduction pathway [8]. Betulin, isolated from *H. syriacus* [9], is a natural triterpene that has anti-cancer effects, but its mechanism of action remains unclear [10,11].

*H. syriacus* is found in tropical and subtropical areas. The flower, fruit, root, stem, and skin of *H. syriacus* all show pharmaceutical effects and have been widely used as medicinal treatment materials in Asia. The flower from *H. syriacus* is used for the treatment of dysentery, vaginal discharge, and hemorrhoids; the fruit is used as an expectorant and for cough and neurological headache; the skin has antipyretic, anthelmintic, antibacterial effects, and its oral administration is used for the treatment of dysentery and vaginal discharge, while external application is used for the treatment of eczema, psoriasis, and scabies [12,13]. In 1998, Yoo *et al.* discussed the components of *H. syriacus* skin in depth [14]. However, there are few references discussing its anti-cancer effects. Only a study in 2008 by Cheng *et al.* showed that *H. syriacus* skin extract activated p53 and apoptosis-inducing factor (AIF), which induced apoptosis in human lung cancer cells [15].

In this study, fifteen *H. syriacus* skin extracts were screened, including seven crude extracts and eight pure compounds. After treating estrogen receptor (ER)-negative and triple-negative breast cancer cell lines with the extracts, functional assays were performed, which showed cell viability-inhibitory effects. In addition, triterpenoids (betulin and its derivatives) isolated from *H. syriacus* skin activated the signaling pathway regulated by p53 family genes, leading to the inhibition of breast cancer cell viability or even the induction of apoptosis. And those triterpenoids had no effect on normal breast cells. These findings provide an important basis for the use of those triterpenoids in the development of alternative therapies for breast cancer treatment.

## Methods

### Cell lines

Human breast cancer cell lines MDA-MB-231 and HBL-100 were originally obtained from American Type Culture Collection (ATCC, Manassas, VA). Non-tumorigenic human breast epithelial cell line H184B5F5/M10 was obtained from the Bioresource Collection and Research Center (BCRC, Taiwan). MDA-MB-231 and HBL-100 were maintained in Dulbecco’s Modified Eagle medium (DMEM) containing 10% fetal bovine serum, 1 μg/ml penicillin and 1 μg/ml streptomycin (Invitrogen) at 37°C in a 5% CO_2_ atmosphere. H184B5F5/M10 cells were grown in MEM-α with the same supplements and culture condition.

### Plant material and compounds

The root bark of *H. syriacus* was obtained from Chien-Yuan Co., Taipei, Taiwan, in September 2009, and the plant was authenticated by Hang-Ching Lin from a voucher specimen (NDMCP no. 980901). All compounds for screening were offered by Professor Wen-Liang Chang, and the detailed isolating approaches of those compounds are described in reference [16].

### Cell viability determined by MTT assay

Cells treated with the indicated concentrations of compounds were washed twice with phosphate buffered saline (PBS) and subjected to the 3-(4, 5-Dimethylthiazol-2-yl)-2, 5-diphenyl-tetrazolium bromide (MTT) assay to measure proliferation. In brief, 20 μl of 5 mg/ml MTT reagent was added to each well and incubated at 37°C for 3.5 h before reading absorbance at 570 nm. A_570_ was recorded at 0 h, 24 h, 48 h, and 72 h after treatment. Each condition was performed in six replicates. Cell morphology was visualized under the indicated conditions by using an Olympus CKX41 light microscope.

### Migration assays

Wound healing assays were employed to evaluate the effects of those compounds on the cell migratory ability. Cells were plated in 6-cm dishes and cultured to >90% confluence. They were scraped with a p200 pipette tip (time 0), transferred to low-serum culture medium, and treated as indicated. The distances of migrating cells were measured from pictures (five fields) taken at 0h, 12h, and 24h, and the distance of each measurement was calculated by ImageJ (NIH, USA). Each experiment was independently repeated at least three times.

### Western blot analysis

Treated cells were washed twice with PBS, then lysed in 200 μl of RIPA lysis buffer (Millipore, 50 mM Tris–HCl, pH 7.4, 150 mM NaCl, 1 mM EDTA, 1% Triton X-100, 1% sodium deoxycholate, 0.1% SDS) containing protease inhibitor (Roche). Thirty micrograms of protein from the supernatant was loaded on an SDS-polyacrylamide gel, followed by western blot analysis (antibody information is listed in Additional file 1: Table S2). The immuno-reactive bands were revealed by an ECL system (Millipore) then developed and quantified on a UVP BioSpectrum Imaging System. Each band was quantified using ImageJ. β-Actin used as internal (loading) control for relative quantitation.

### Data analysis

Real-time PCR original data, western blot data and wound healing regions were recorded as continuous variables and analyzed with Student’s *t*-test. All the statistical analyses were performed using SPSS 16.0 and Excel 2010. All statistical tests and *P* values were two-sided. The level of significance was set at *P* < 0.05.

### Real-time PCR and flow cytometry

Detail methods of RNA preparation, quantitative real-time PCR, and flow cytometry were described in Additional file 1.

## Results

### Toxicity analysis of *H. syriacus* extracts on breast cancer cell lines

**Figure 1:**
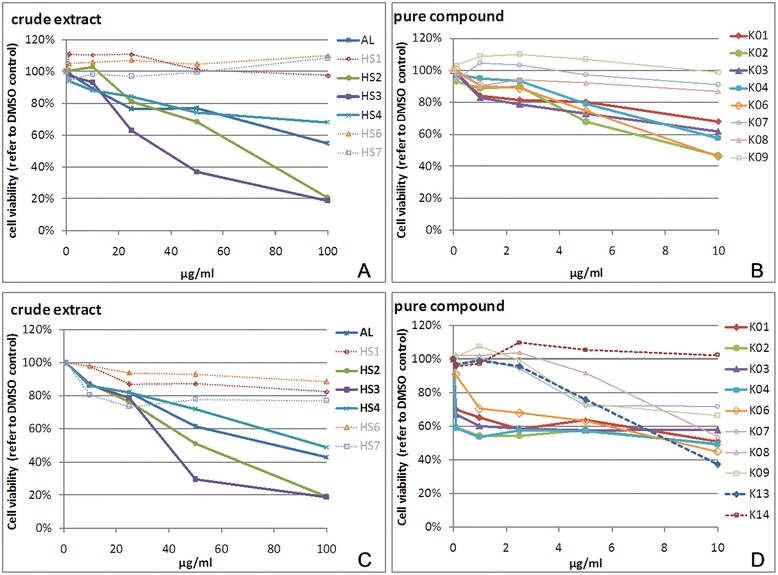
**The effect of** ***H. syriacus*** **skin crude extracts and pure compounds on the cell viability of MDA-MB-231 and HBL100 cell lines.** MDA-MB-231**(A)** and HBL100 **(B)** were treated with 0, 1, 10, 25, 50, or 100 μg/mL crude extracts for 48 h and subjected to MTT assay to analyze the cell viability of MDA-MB-231 and HBL100 cell lines. **(A)** MDA-MB-231 cells treated with 100 μg/ml HISY-F2 or HISY-F3 showed 20% cell viability compared with DMSO controls (0.1% DMSO). MDA-MB-231 cells treated with 50 μg/mL of HISY-F3 crude extract showed a cell viability of <50% compared with DMSO controls. **(B)** Cell viability of HBL-100 cells treated with 50 μg/mL of HISY-F2 or HISY-F3 was 20% of DMSO controls, and the cell viability of HBL-100 cells treated with 100 μg/mL of HISY-F2 or HISY-F3 was less than 20% of that of DMSO controls. **(C)** Cell viability decreased by 5 μg/mL treatment with K01, K02, K03, K04, or K06 compared with DMSO controls; 10 μg/mL of K02 or K06 reduced cell viability to <50% compared with DMSO controls. **(D)** Cell viability decreased by 1 μg/mL treatment with K01, K02, K03, K04, or K06 compared with DMSO controls; 10 μg/mL of K01, K02, K03, K04 or K06 reduced cell viability to <50% compared with DMSO controls.

Seven crude extracts (100, 50, 25, 10, 1 μg/ml) and eight pure compounds (10, 5, 2.5,1, 0.1 μg/ml) from *H. syriacus* were used to treat invasive MDA-MB-231 and noninvasive HBL-100 breast cancer cell lines for 48 h before an MTT assay was performed. HISY-F2, HISY-F3, and HISY-F4 showed the best effects on both breast cancer cell lines. The effects of these pure compounds on breast cancer cell viability varied, including a poor inhibitory effect of K07, K08, and K09 and a strong inhibitory effect of K01, K02, K03, and K04. The inhibitory effect of K06 was the strongest (Figure 1). In addition, based on the original analysis of the pure compounds, the inhibition of cell viability by the pure compound was consistent with that of the crude extract. On the other hand, MDA-MB-231 cells showed a stronger tolerance to drug treatment. Thus, the inhibitory effect on MDA-MB-231 was poorer than that on HBL-100 cells (Figure 1).

The results from the MTT assay showed that the inhibitory effects of K01, K02, K03, K04, and K06 on the cell viability of breast cancer cell lines were the greatest. However, they had non-significant effects on the cell viability of the non-tumorigenic human breast epithelial cell line H184B5F5/M10 (Additional file 1: Figure S5E). Compound structural analysis showed that K01, K02, K03, K04, and K06 were the derivatives of betulin (Additional file 1: Figure S1), but the mechanism of action of these compounds was unclear. Thus, we evaluated the biological effects of betulin and its derivatives on breast cancer cells.

### Betulin and its derivatives induce morphology changes in breast cancer cell lines

**Figure 2:**
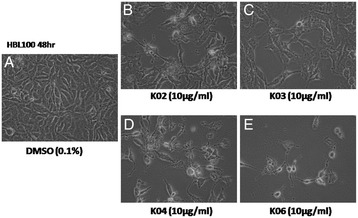
**Betulin and its derivatives cause morphological changes in HBL100 cells.** After treating HBL-100 cells with 10 μg/mL of K02 **(B)**, K03 **(C)**, K04 **(D)** or K06 **(E)** for 48 h, cell morphology changed to star-shaped and linear-shaped with vacuolation, and cell density significantly decreased. In the DMSO control group (**A**, 0.1% DMSO), cell shapes were intact and flat with higher density.

After treating HBL-100 cells with 10 μg/mL of K02, K03, K04, or K06 for 48 h, cell morphology was examined under microscopy. In the DMSO control group, the cells were flat and adhered to the culture dish with high cell density. However, the cells treated with those triterpenoids became long, slender, and spindle-and star-shaped. In addition, the nucleus showed vacuolization, and the cell density decreased (Figure 2). Thus, betulin and its derivatives inhibited breast cancer cell viability and may have induced breast cancer cell apoptosis.

### Betulin and it derivatives induce breast cancer cell apoptosis

To examine the cell cycle and proportion of sub-G1 phase cells in breast cells after treatment with betulin or its derivatives, HBL-100 and H184B5F5/M10 cells were treated with 10 μg/mL of K02, K03, K04 or K06 for 48 h, stained with PI and subjected to flow cytometry. The cells treated with those triterpenoids showed a significant increase in the proportion of cells in sub-G1 phase compared to the DMSO control group. These results indicate that betulin and it derivatives induced apoptosis in breast cancer cells (Additional file 1: Figure S2). The compounds had a relatively lower toxicity to the non-tumorigenic human breast epithelial cell line H184B5F5/M10 (Additional file 1: Figure S5F).

HBL-100 cells treated with 10 μg/mL of K02, K03, K04 or K06 for 48 h were further double-stained with Annexin V/PI for flow cytometry to determine the toxic effects of betulin and its derivatives on breast cancer cells. The results showed that >50% (the sum of the first and fourth quadrant) of cells were apoptotic after K06 treatment, approximately 40% were apoptotic after K02 treatment, and approximately 25% of cells were apoptotic after K03 or K04 treatment. Overall, apoptosis was significantly increased in the drug-treated groups compared with DMSO control cells (2.06% apoptotic cells) (Additional file 1: Figure S3). And K02 and K06 possessed a greater ability to induce apoptosis than K03 and K04.

### Betulin and it derivatives inhibit migration of breast cancer cells

**Figure 3:**
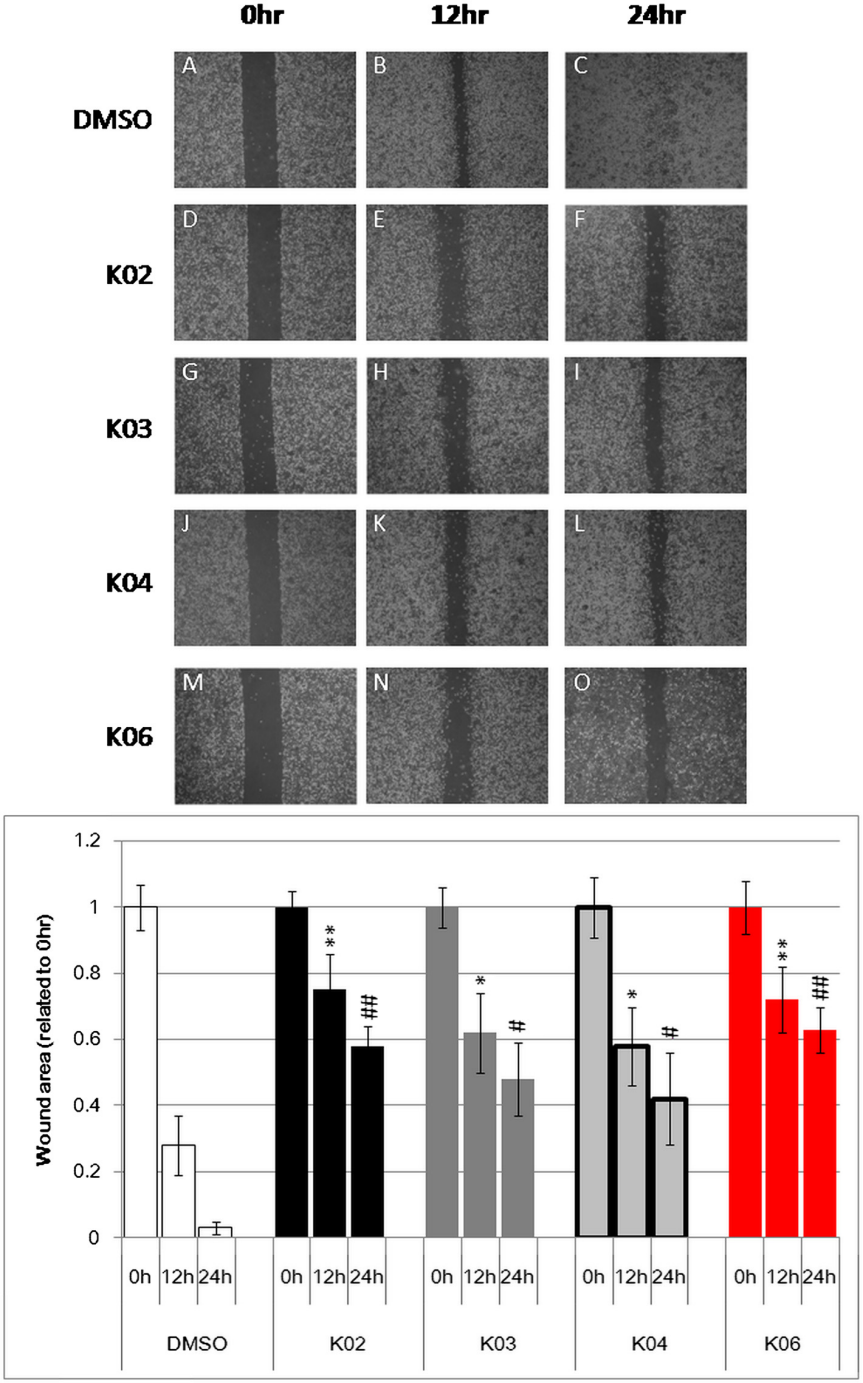
**Betulin and its derivatives inhibit cell migration in the MDA-MB-231 cell line.** A wound healing assay was performed to determine the cell migration ability. MDA-MB-231 cells were treated with 0.1% DMSO **(A, B, C)** or 5 μg/mL K02 **(D, E, F)**, K03 **(G, H, I)**, K04 **(J, K, L)**, or K06 **(M, N, O)** for 24 h. The cells were observed every 12 h under the same observation field for wound healing status. At 12 h, cell migratory abilities in K03- **(H)** and K04-treated **(K)** cells slightly decreased compared with DMSO controls **(B)**. In K02- **(F)** and K06-treated **(N)** cells, cell migratory abilities more obviously decreased compared with DMSO controls **(B)**. At 24 h, cell migratory abilities in the K02 **(L)**, K03 **(H)**, K04 **(K)**, and K06 **(O)** groups significantly decreased compared with DMSO controls **(B)**, and the wound area was larger than that of DMSO controls **(C)**. The wound area was quantified by ImageJ software. Compared with 12 h of DMSO treatment, the wound area was slightly significantly larger (* p < 0.05) in the K03- and K04-treated groups and more significantly larger in the K02- and K06-treated groups (** p < 0.01). Compared with 24 h of DMSO treatment, the wound area was significantly larger (# p < 0.05) in the K03- and K04-treated groups and also significantly larger in the K02- and K06-treated groups (## p < 0.01).

A wound healing assay was performed to observe the effect of betulin and its derivatives on the migration ability of breast cancer cells. After treating invasive MDA-MB-231 cells with 5 μg/mL (about IC_25_was used to reduce the interference of apoptosis) of K02, K03, K04,or K06 for 24 h, wound healing status was determined under the same field every 12 h. In the DMSO control group, the wound area was almost healed at 24 h. The wound areas of K02-, K03-, K04-, or K06-treated cells were significantly larger at 12 h and 24 h than DMSO controls (Figure 3 upper). After quantifying the wound areas with ImageJ, the results showed that this region shrank faster in the DMSO control compared to the K02-, K03-, K04-, or K06-treated groups. Thus, betulin and it derivatives inhibited the migratory ability of breast cancer cells, especially K02 and K06 (Figure 3 lower).

### Betulin and its derivatives induce apoptosis in breast cancer cells was related to TAp63

The *H. syriacus* skin extracts K02 and K06 induced apoptosis in MDA-MB231 cells. Betulin and betulinic acid can induce cancer cell death via intrinsic apoptotic pathways [17,18]. Furthermore, *H. syriacus* skin extracts activate p53 and AIF, leading to lung cancer cell apoptosis [15]. After treating cells with 10 μg/mL of K02, K03, K04 or K06 for 12, 24, or 36 h, we performed real-time PCR to analyze the apoptosis-related genes BAX, NOXA, PERP, and PUMA, and the p63 isoforms ΔNp63 and TAp63. p63 is one member of the p53 tumor suppressor gene family, and it is highly similar to p53 in protein structure. Based on the sequence at the N-terminal end of the protein, p63 is classified into two isoforms, ΔNp63 and TAp63 [19,20]. ΔNp63 and TAp63 antagonize each other in the regulation of gene expression. For example, ΔNp63 inhibits TAp63-regulated p53 downstream gene expression [19,20] and promotes anti-apoptotic gene expression. After treating with K02 and K06 for 12 h, TAp63 was significantly increased at 24 h and slightly decreased at 36 h. The TAp63 antagonist ΔNp63 also increased at 12 h and dramatically decreased at 24 h or 36 h. In addition, most of the TAp63 downstream apoptotic genes, including BAX, NOXA, PUMA and PERP, increased over time (Additional file 1: Figure S4A and 4D). These results imply that K02 and K06 induced apoptotic gene expression is TAp63 associated in the MDA-MB-231 cell line. Although K03 and K04 treatment also increased TAp63 expression, no significant change was observed in ΔNp63 or downstream apoptotic genes (Additional file 1: Figure S4B and 4C). Therefore, in p53-mutated MDA-MB-231 breast cancer cells, K02 and K06 may induce TAp63 expression to compensate for parts of p53 function.

**Figure 4:**
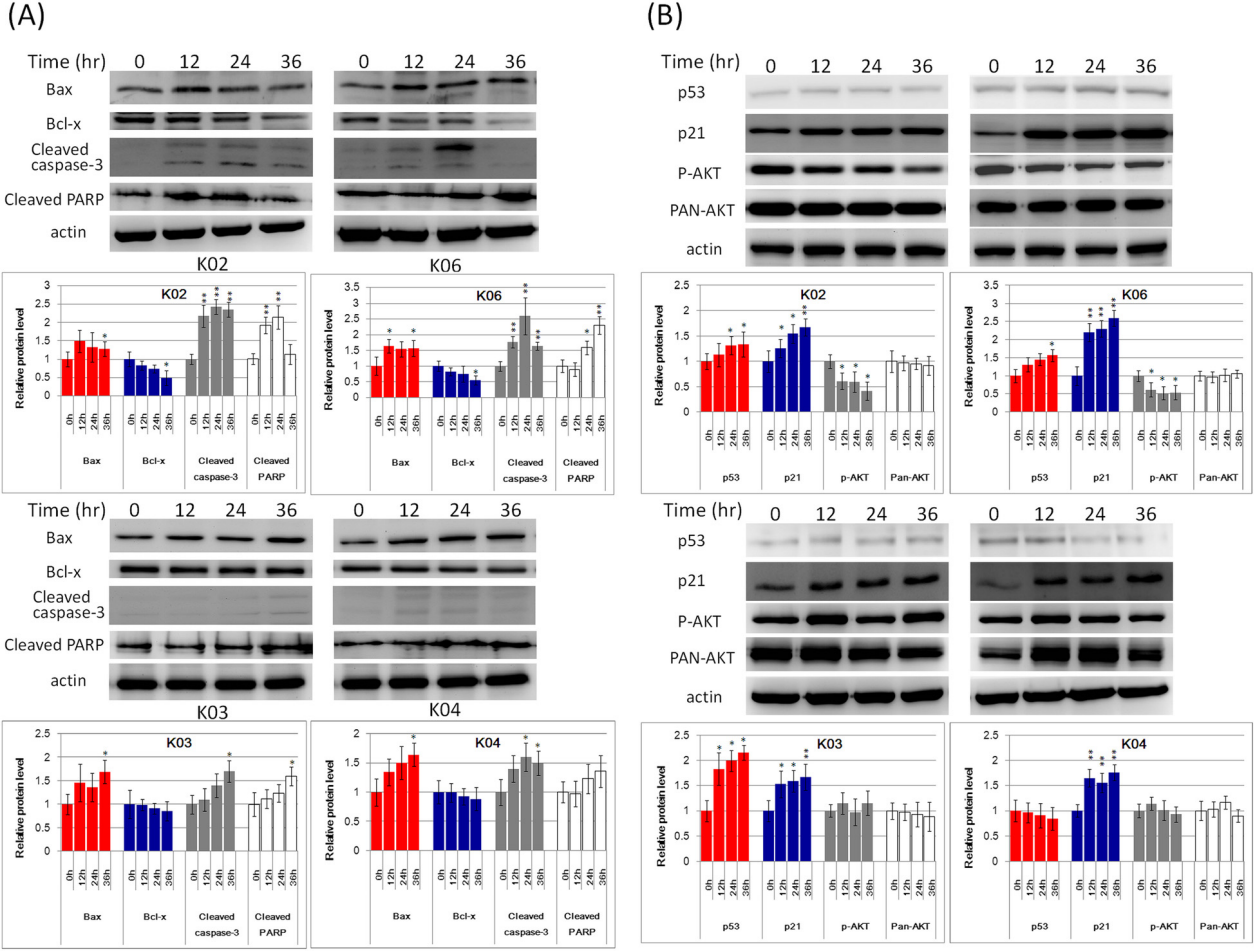
**K02, K03, K04, and K06 induce the expression of apoptosis-related genes in the HBL-100 cell line. (A)** After treating HBL-100 cells with 10 μg/mL of K02, K03, K04, or K06 for 0, 12, 24, or 36 h, western blot was performed to analyze apoptosis-related proteins. β-Actin was used as the internal control for relative quantitation. Cleaved caspase-3 increased with treatment time to a peak at 24 h and decreased at 36 h under K02 and K06 treatment. Cleaved PARP also increased with treatment time to a peak at 24 h under K02 and K06 treatment. Bax expression was significantly increased at 36 h with all four compounds. Bcl-x expression decreased with treatment time, though without significance.**(B)** After treating HBL-100 cells with 10 μg/mL of K02, K03, K04, or K06 for 0, 12, 24, or 36 h, western blot was performed to analyze p53, p21, p-AKT and total AKT. p53 and p21 expression were induced after treatment with K02, K03 or K06 and increased with treatment time with all four compounds. p-AKT progressively decreased after treatment with K02 and K06, while total AKT showed no changes.

After treating HBL-100 cells with 10 μg/mL of K02, K03, K04 or K06 for 12, 24, or 36 h, the pro-apoptotic protein Bax significantly increased at 36 h, and the anti-apoptotic gene Bcl-x slightly decreased over time, though without significance. Cleaved caspase-3 and cleaved PARP gradually increased after treated with those triterpenoids; K02 and K06 induced significantly higher cleaved caspase-3 after 12 h and higher cleaved PARP at 36 h (Figure 4A). Furthermore, K02, K06, K03 and K04 treatment induced higher p53 and its downstream protein p21, whereas p-AKT was downregulated. Especially, K02 and K06 significantly induced p53 and p21 and reduced p-AKT (Figure 4B). These results indicate that betulin and its derivatives induced HBL-100 cell apoptosis by increasing Bax, cleaved caspase-3 and cleaved PARP and decreasing Bcl-x; and they inhibited HBL-100 cell growth by terminating the cell cycle by increasing p53 and p21 and decreasing AKT phosphorylation (Figure 4). K02 and K06 showed stronger activity for the induction of apoptotic and cell cycle inhibitory genes than the other compounds. In the MDA-MB231 cell, the p53 gene was mutated. Thus, it was necessary to clarify the molecular mechanism how those triterpenoids induced apoptosis in this cell line. It is evident that p53-related apoptotic genes are also regulated by p63 [21], and therefore p63 may participate in those triterpenoids induced apoptosis in MDA-MB-231 cell.

Real-time PCR was performed to analyze ΔNp63 and TAp63 RNA expression. After treatment with K02 or K06 for 12 h, TAp63 significantly increased at 24 h and slightly decreased at 36 h. ΔNp63 also increased at 12 h, but it lost significance or returned close to baseline at 24 h or 36 h. Although K03 and K04 treatment also increased TAp63 expression, ΔNp63 expression showed no significant changes. These results indicate that in the MDA-MB-231 cell, K02 and K06 induced apoptotic gene expression in a TAp63-associated manner. Although K03 and K04 treatment increased TAp63 expression, no increase in downstream gene expression was observed (Additional file 1: Figure S4). Thus, in p53-mutated MDA-MB-231 breast cancer cells, K02 and K06 may induce TAp63 expression to compensate parts of p53 function. In the non-tumorigenic human breast epithelial cell, H184B5F5/M10, the expression of BAX, NOXA, PUMA and PERP showed no change with K02, K03, K04 or K06 treatment, whereas TAp63 decreased with treatment time and ΔNp63 expression fluctuated (Additional file 1: Figure S5A-D).

## Discussion

Accumulating evidence demonstrates that induction of apoptosis in cancer cells is the major effect of chemotherapy [9,15]. In this study, betulin and its derivatives also induced human breast cancer cell apoptosis, and there were different susceptibilities between the cell lines. For instance, betulin (K02) and betulinic acid (K06) led to higher apoptosis in HBL-100 than MDA-MB-231 cells (Figure 1C and 1D). Dysregulated apoptosis is typically observed in cancer cells; however, betulin derivatives regulated different apoptotic proteins in different cancer types [10,11,17]. In the present study, betulin (K02) and betulinic acid (K06) had different IC_50_ values between HBL-100 and MDA-MB-231 cells (Figure 1A and 1B). Because MDA-MB-231 is a p53-mutant cell line and HBL-100 is a p53-wild-type cell line, we found that MDA-MB-231 cells induced the tumor suppressor TAp63 in a compensatory manner. TAp63 is a highly conserved member of the p53 family that is structurally similar to p53; therefore, it was able to induce apoptosis by activating p53 downstream target genes, including BAX, NOXA, PUMA, and PERP (Additional file 1: Figure S4) [21-23]. TAp63 can also induce p21 expression and has been associated with cell cycle arrest and apoptosis [24,25].

In addition, K03 and K04 betulin derivatives induced a lower level of apoptosis than K02 and K06 (Additional file 1: Figure S2); however, K03 and K04 caused G2/M arrest, as shown by flow cytometry. Furthermore, K03 and K04 repressed cell migration in wound healing assays (Figure 3).

Basal-like breast cancer is generally triple-negative (ER-negative, PR-negative, and Her2/neu-negative), with a similar gene expression profile to myoepithelial cells [26]. However, it remains difficult to clearly distinguish basal-like breast cancer from triple-negative breast cancer [27]. Clinically, triple-negative breast cancer possesses higher invasiveness, more distal metastasis and poor prognosis [28]. The BRCA1 tumor suppressor participates in DNA repair and is often mutated in triple-negative breast cancers [29]. The basal-like breast cancer gene expression profile is also similar to BRCA1-mutated breast cancer, as shown by microarray [30] and immunohistochemical analysis [31], particularly in early-onset or familial breast cancer, and it is usually accompanied by a p53 mutation. Several studies have addressed the association between p53 and anthracycline treatment [32,33], and p53 functions as a prognostic indicator of triple-negative breast cancer therapeutic efficacy [34]. We employed two triple-negative breast cancer cell lines, MDA-MB-231 and HBL100 [35], to investigate the regulatory mechanism of *H. syriacus* extracts on cell proliferation and apoptosis. Therefore, *H. syriacus* extracts or betulin and its derivatives may function as potential adjuvant treatments in p53-mutant cancers [36,37].

## Conclusions

In this study, we have described four major findings: (1) the natural compound betulin (K02) and its derivatives (K03, K04, K06) induced human breast cancer cell apoptosis and inhibited cell proliferation; (2) K02 (betulin) and K06 (betulinic acid) induced apoptotic protein expression, including BAX, NOXA, PUMA and PERP, and also increased caspase-3 cleavage as well reduced the anti-apoptotic gene Bcl-x in MDA-MB-231 and HBL-100 cells; (3) K02 and K06 induced TAp63 in the p53-mutant MDA-MB-231 cell line, leading top53-related apoptosis; (4) betulin and its derivatives induced p21 activation and promoted cell cycle arrest.

## Additional file

Additional file 1
**Figure S1.** Isolation of *H. syriacus* skin extracts. **Figure S2.** Betulin and its derivatives increased the sub-G1 population of HBL-100 cells. **Figure S3.** Betulin and its derivatives induced apoptosis in HBL-100 cells. **Figure S4.** Betulin and its derivatives regulated apoptotic-related gene mRNA expression in MDA-MB-231 cells. **Figure S5.** The effects of betulin and its derivatives on cell viability, apoptosis and apoptotic-related gene expression in non-tumorigenic human breast epithelial cell H184B5F5/M10. **Table S1.** Primer list of real time PCR. **Table S2.** Antibodies list.
