# Supplementary material for: The triterpenoids of Hibiscus syriacus induce apoptosis and inhibit cell migration in breast cancer cells
Source: BMC Complement Altern Med. 2015 Mar 14;15:65. doi: 10.1186/s12906-015-0592-9 (PMC4410586; doi:10.1186/s12906-015-0592-9)
Supplement: Additional file 1 — Figure S1. Isolation of H. syriacus skin extracts. Figure S2. Betulin and its derivatives increased the sub-G1 population of HBL-100 cells. Figure S3. Betulin and its derivatives induced apoptosis in HBL-100 cells. Figure S4. Betulin and its derivatives regulated apoptotic-related gene mRNA expression in MDA-MB-231 cells. Figure S5. The effects of betulin and its derivatives on cell viability, apoptosis and apoptotic-related gene expression in non-tumorigenic human breast epithelial cell H184B5F5/M10. Table S1. Primer list of real time PCR. Table S2. Antibodies list. [file 12906_2015_592_MOESM1_ESM.docx]

**Supplemental methods**

**RNA preparation and quantitative real-time PCR**

Total RNA of treated cells was isolated by TRIzol (Invitrogen). RNA samples were treated with the RQ1 RNase-free DNase (Promega) to remove any genomic contamination according to the manufacturer’s instructions. Five micrograms of treated RNA samples was subjected to reverse transcription with SuperScript III (Invitrogen). Quantitative real-time PCR was processed by StepOne Real-Time PCR System (Applied Biosystems) using Maxima Hot Start PCR Master Mix (2×) (Fermantus), and GAPDH was used as an internal control. Besides melting curve, real time PCR products were also analyzed by gel electrophoresis to confirm single PCR products. Primer sets were listed in supplemental table 1.

**Flow cytometry**

Subconfluent cells were trypsinized, washed with PBS. A total of 1 x10^6^ cells were fixed with 100% ethanol for 10 min following incubated with 1 mg/ml propidium iodide (PI) for 10 min at room temperature. Cells were analyzed within 20 min post-staining on a BD FACSCalibur (BD Biosciences). Quantitation of different cell cycle phases were categorized by PI-staining intensity as sub-G1 (<800), G1 (800-1200), S (1201-1700), and G2/M (1701-2100). For Annexin V-PI double staining, treated cells were harvested at indicated time, wash twice with ice cold PBS, and directly stained with FITC-conjugated Annexin V and PI (BD Biosciences) for 15 min without fixation. Quadrant was divided as x-axis: Annexin-positive (FITC intensity >=45) and Annexin-negative (FITC intensity <45), and y-axis: PI-positive (PI intensisty >=40) and PI-negative (PI intensity <40).

Figure S1

(A)


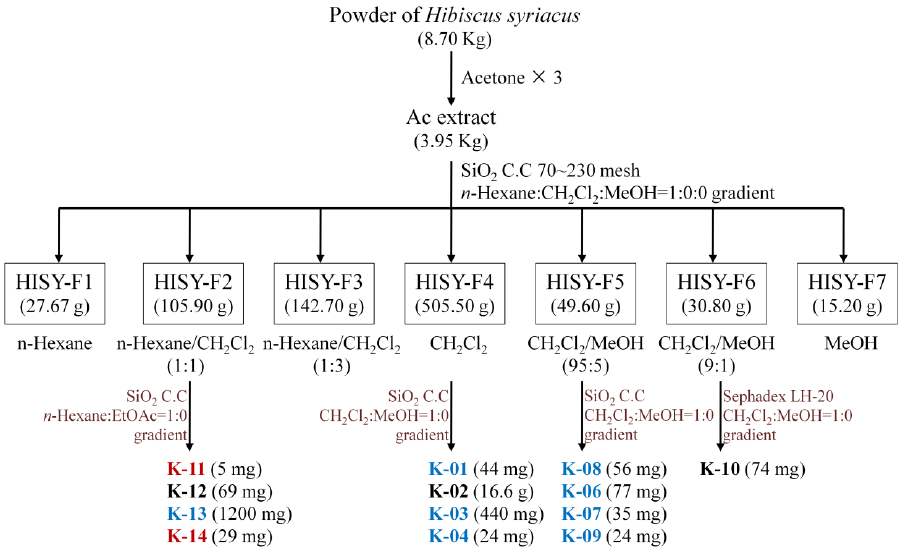


(B)

Figure S1. Isolation of *H. syriacus* skin extracts

Root skin powder from *H. syriacus* (8.70 kg) was rinsed three times with non-polar organic solvent acetone, and the acetone layer was obtained. Following by increasing the polarity of organic solvent to isolate acetone extract, and the crude extracts HISY-F1 (*Hibiscus syriacus*), HISY-F2, HISY-F3, HISY-F4, HISY-F5, HISY-F6 and HISY-F7. Subsequently, the polarity of organic solvent increased again, and the pure compounds K01-K14 were extracted from HISY-F2, HISY-F4, HISY-F5 and HISY-F6.

Figure S2

Figure S2. Betulin and its derivatives increased the sub-G1 population of HBL-100 cells

After treating HBL-100 with 0.1% DMSO (A), or 10 µg/mL of K02 (B), K03 (C), K04 (D) or K06 (E) for 48 h, the cells were stained with PI for flow cytometry. Compared to DMSO control (A), HBL-100 cells treated with K02, K03, K04, and K06 showed a significant increase in sub-G1 population, in particular the cells treated with K02 (B) and K06 (E). Quantitation of cells in sub-G1, G1, and G2/M phases showed that over 50% of cells were in sub-G1 phase in cells treated with K02 and K06.

Figure S3

Figure S3. Betulin and its derivatives induced apoptosis in HBL-100 cells.

After treating HBL-100 with 10 µg/mL of K02 (B), K03 (C), K04 (D) and K06 (E) for 48 h, the cells were double stained with FITC-conjugated Annexin V and PI for flow cytometry. The results were quantified for each quadrant. Compared to DMSO control (0.1% DMSO) (A), cells in quadrant I and IV increased in cells treated with K02, K03, K04 and K06, especially in cells treated with K02 (B) and K06 (E). After quantifying all four quadrants, cell number in quadrant I and IV from K02 and K06 treated groups was more remarkably increased whilst compared to those of K03 and K04 treated and DMSO control groups (F).

Figure S4.

Figure S4. Betulin and its derivatives regulated apoptotic-related gene mRNA expression in MDA-MB-231 cells.

After treating MDA-MB-231 with 10 µg/mL of K02 (A), K03 (B), K04 (C) or K06 (D) for 0, 12, 24 and 36 h, real-time PCR was performed to analyze TAp63, ΔNp63, BAX, PUMA, NOXA and PERP mRNA expression. Among K02 and K06 treated cells, TAp63 was significantly increased at 12 h and slightly decreased at 24 h and 36 h. ΔNp63 also increased at 12 h, and it lost significance and dramatically decreased at 24 h or 36 h. And most of TAp63 downstream apoptotic genes including BAX, NOXA, PUMA and PERP were increased over time (A and D). These results implicated that K02 and K06 induced apoptotic gene expression in a TAp63-associated manner. Although K03 and K04 treatment also increased TAp63 expression, no significant change was observed in ΔNp63 or downstream apoptotic genes (B and C). Therefore, in p53-mutated MDA-MB-231 breast cancer cells, K02 and K06 may induce TAp63 expression to compensate parts of p53 function.

Figure S5.

1. MTT assay

1. Flow cytometry

Figure S5. The effects of betulin and its derivatives on cell viability, apoptosis and apoptotic-related gene expression in non-tumorigenic human breast epithelial cell H184B5F5/M10.

After treating H184B5F5/M10 with 10 µg/mL of K02 (A), K03 (B), K04 (C) or K06 (D) for 0, 12, 24 and 36 h, real-time PCR was performed to analyze TAp63, ΔNp63, BAX, PUMA, NOXA and PERP mRNA expression. Among all treated cells, the expression of BAX, NOXA, PUMA and PERP was not changed. In, TAp63 expression increased first and decreased afterwards in K02 or K06 treated cells. ΔNp63 increased first and decreased afterwards in K02 treated cells and fluctuated in K06 treated cells. In K03 and K04 treated cells, ΔNp63 was not obviously changed, and the expression of TAp63 increased first and decreased over time. Besides, H184B5F5/M10 cells were treated with 0.1% DMSO, or 10 µg/mL of K02, K03, K04 or K06 for 48 h and harvested for (E) MTT assay and (F) PI-staining flow cytometry. The detail methods were identical as described in text, legend of Figure 1, and supplemental methods. Betulin and its derivatives were not obviously modified cell viability and apoptosis of H184B5F5/M10 cells and therefore implicated a relative lower toxicity to normal mammary cells.

Supplemental table 1 Primer list of real time PCR

|  | Forward primer | Reverse primer |
| --- | --- | --- |
| TAp63 | CAGTCCAGAGGTTTTCCAGCAT | TCAATGGGCTGAACATATAG |
| ΔNp63 | GCAAAACAATGCCCAGACTCA | TGTTCAGGAGCCCCAGGTT |
| BAX | ATGTTTTCTGACGGCAACTTC | ATCAGTTCCGGCACCTTG |
| PUMA | ACCTCAACGCACAGTACGA | GAGATTGTACAGGACCCTCCA |
| NOXA | GGAGATGCCTGGGAAGAAG | CCTGAGTTGAGTAGCACACTCG |
| PERP | TGTCTTCCTGAGAGTGATTGGA | ACCAGGGAGATGATCTGGAA |
| GAPDH | CCACTCCTCCACCTTTGAC | ACCCTGTTGCTGTAGCCA |

Supplemental table 2 Antibodies list

|  | Primary Antibody | Secondary Antibody |
| --- | --- | --- |
| Bax | Rabbit polyclonal Ab  (Santa cruz) | HRP-conjugated Goat anti-rabbit IgG Ab  (Jackson) |
| Bcl-x | Rabbit polyclonal Ab  (Santa cruz) | HRP-conjugated Goat anti-rabbit IgG Ab  (Jackson) |
| Caspase-3 | Rabbit polyclonal Ab  (Cell signaling) | HRP-conjugated Goat anti-rabbit IgG Ab  (Jackson) |
| PARP | Rabbit polyclonal Ab  (Cell signaling) | HRP-conjugated Goat anti-rabbit IgG Ab  (Jackson) |
| p53 | Mouse monoclonal Ab  (Dako) | HRP-conjugated Goat anti-mouse IgG Ab  (Jackson) |
| p21 | Mouse monoclonal Ab  (Santa cruz) | HRP-conjugated Goat anti-mouse IgG Ab  (Jackson) |
| Phospho-AKT (Ser473) | Rabbit polyclonal Ab  (Cell signaling) | HRP-conjugated Goat anti-rabbit IgG Ab  (Jackson) |
| Pan-AKT | Rabbit polyclonal Ab  (Cell signaling) | HRP-conjugated Goat anti-rabbit IgG Ab  (Jackson) |
| β-actin | Mouse monoclonal Ab  (Thermo SCIENTIFIC) | HRP-conjugated Goat anti-mouse IgG Ab  (Jackson) |
